# Over-diagnosis of potential malignant behavior in MEN 2A-associated pheochromocytomas using the PASS and GAPP algorithms

**DOI:** 10.1007/s00423-018-1679-9

**Published:** 2018-05-19

**Authors:** Adam Stenman, Jan Zedenius, Carl Christofer Juhlin

**Affiliations:** 1 Department of Oncology-Pathology, Karolinska Institutet, CCK, Karolinska University Hospital, R8:04, 171 76, Solna, Stockholm Sweden; 2 Department of Molecular Medicine and Surgery, Karolinska Institutet, Stockholm, Sweden; 3 Department of Breast, Endocrine Tumours and Sarcoma, Karolinska University Hospital, Stockholm, Sweden; 4 Department of Pathology and Cytology, Karolinska University Hospital, Stockholm, Sweden

**Keywords:** Pheochromocytoma, MEN 2A, PASS, GAPP, Malignancy

## Abstract

**Purpose:**

Pheochromocytomas (PCCs) exhibit malignant potential, but current histological modalities for the proper detection of aggressive behavior are debated. The two most widespread algorithms are the “Pheochromocytoma of the Adrenal Gland Scaled Score” (PASS) and the “Grading System for Adrenal Pheochromocytoma and Paraganglioma” (GAPP), both which mostly rely on histological parameters to identify PCC patients at risk of disseminated disease. Since the algorithms are derived from studies using predominantly sporadic PCCs, little is known whether the PASS or GAPP scores can predict malignant potential in hereditary cases.

**Methods:**

PASS and GAPP were applied on 41 PCCs; 13 PCCs were diagnosed in ten multiple endocrine neoplasia type 2A (MEN 2A) patients carrying established germline *RET* proto-oncogene mutations, as well as 28 assumed sporadic PCCs.

**Results:**

Six out of thirteen MEN 2A tumors (46%) exhibited PASS scores ≥ 4, indicative of a potential for aggressive behavior. In addition, 7/13 tumors (54%) exhibited GAPP scores ≥ 3, indicative of a “moderately differentiated type” with risk of future recurrence. All MEN 2A PCCs with an elevated PASS score also displayed an elevated GAPP score. In contrast, 4/28 (14%) sporadic PCCs demonstrated PASS scores ≥ 4, and 9/28 (32%) displayed GAPP scores ≥ 3. Follow-up found all cases in the study are free of metastatic or recurrent disease.

**Conclusions:**

We conclude that the PASS and GAPP scoring systems might be suboptimal for determining true malignant potential in PCCs with constitutional *RET* mutations and advocate restrictive use of these scores in MEN 2A cases until the results are reproduced in larger numbers.

## Introduction

Pheochromocytomas (PCCs) are rare endocrine tumors derived from the adrenal medulla. Although the majority of tumors are sporadic, a large subset of cases displays a hereditary background [1]. The current WHO criteria from 2017 states that all PCCs are potentially malignant and should be risk stratified using one or both of the established algorithms: the Pheochromocytoma of the Adrenal Gland Scaled Score (PASS) [2] and an alternative scoring system by Kimura et al. (the Grading System for Adrenal Pheochromocytoma and Paraganglioma (GAPP)) [3]. While PASS incorporates a scoring system including various histological parameters, the alternative scoring system GAPP extends on this and includes histological as well as immunohistochemical (Ki-67) and clinical (catecholamine phenotype) variables. When assessing malignant potential of PCCs, the PASS algorithm is probably the most widespread scoring system. A score from 0 to 20 points is acquired based on histological findings, in which tumors with a PASS score ≥ 4 have the potential for aggressive behavior. The GAPP score categorize PCCs into three different types, well-differentiated PCCs (score 0–2), moderately differentiated PCCs (score 3–6), and poorly differentiated PCCs (score 7–10), in which the two latter groups carry an increased risk of recurrences and future metastases.

In our institution, we have empirically noted that MEN 2A PCCs often obtain high PASS and GAPP scores. However, as MEN 2A-related PCCs (as well as PCCs with somatic *RET* mutations) are very seldom malignant [1], this case made us unsure as to whether the PASS and GAPP systems are applicable to this specific subset of PCCs. We therefore sought to determine the PASS and GAPP score ranges and the follow-up for PCCs with established constitutional *RET* mutations at our institution, to investigate whether these classification systems can predict patients at risk for malignancy in MEN 2A patients.

## Materials and methods

**Fig. 1:**
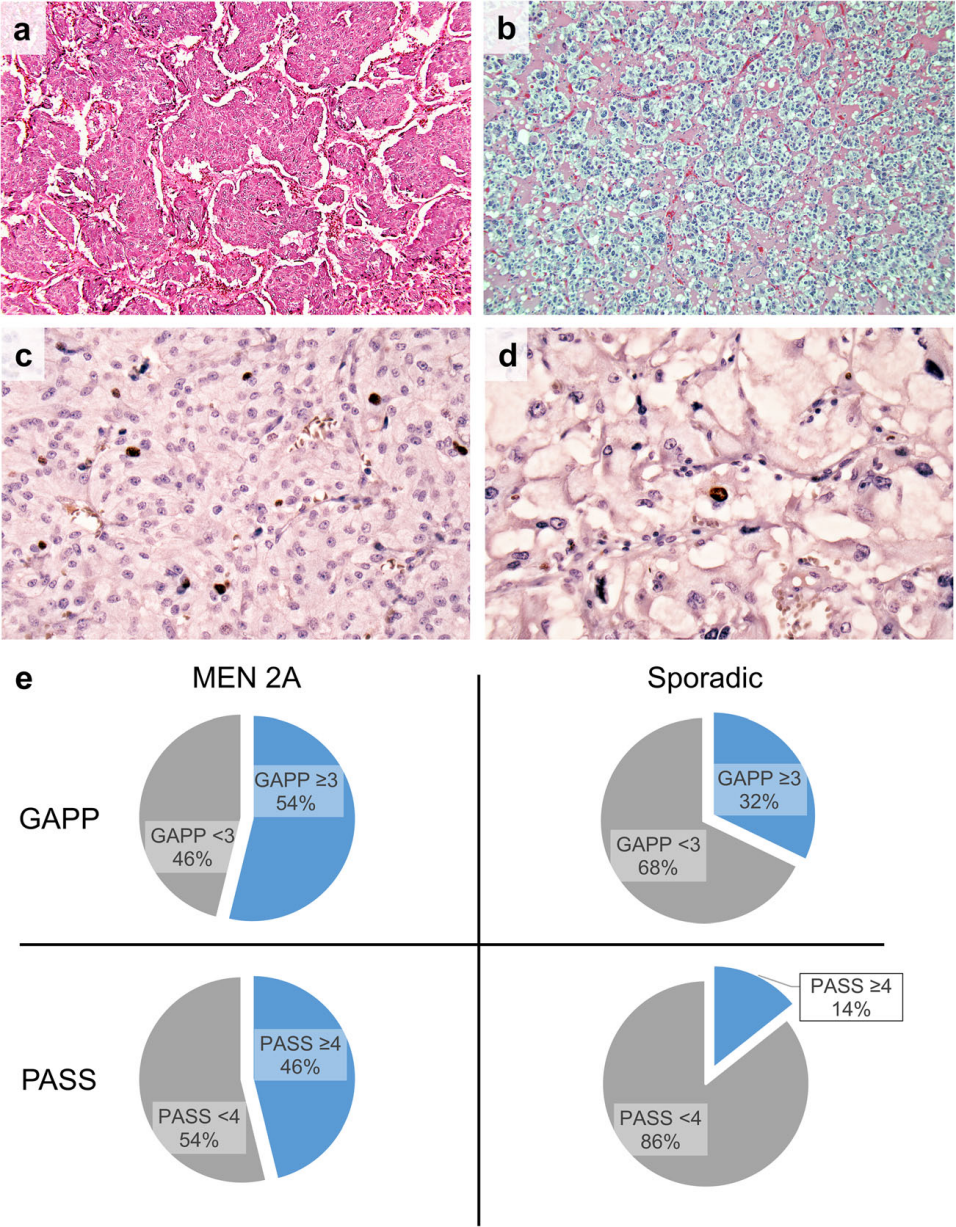
Photomicrographs illustrating frequently observed PASS and GAPP parameters in MEN 2A-associated PCCs. **a** Case 9 (MEN 2A PCC) demonstrating large, irregular nests. This was the most frequently fulfilled PASS criteria and the second most common GAPP criterion in our MEN 2A cohort. Image at × 100 magnification. **b** Case 27 (sporadic control) displaying a classical, alveolar growth pattern (“zellballen”) as comparison. Image at × 100 magnification. **c** Case 4 (MEN 2A PCC) displaying a Ki-67 index of 2.5%. Elevated Ki-67 index was the most frequently observed GAPP criterion among the MEN 2A cases. **d** Case 33 (sporadic control) demonstrating a Ki-67 index of < 1% as comparison. **e** Pie charts showing the ratio of cases with GAPP scores ≥ 3 and PASS ≥ 4 in the group of sporadic PCCs (*n* = 28) and in the MEN 2A group (*n* = 13)

We screened our clinical databases and identified 10 patients with 13 resected PCCs during the period 1987–2017, all patients harboring *RET* constitutional mutations (c.1900T>C in two, c.1858T>C in one, and c.1900T>G in the remaining seven patients) (Table 1). As an assumed sporadic control group, 28 unilateral PCCs diagnosed consecutively at our institution between 2011 and 2017 in patients ≥ 40 years of age with unknown *RET* mutational status but no apparent family history suggesting hereditary disease were included. No paragangliomas were included in the study. An endocrine pathologist (CCJ), blinded to clinical outcome, reviewed the original pathology reports and reviewed the histopathological characteristics of all cases using the PASS and GAPP scoring systems. To be able to fully acquire a GAPP score for each case, patient charts were reviewed for the procurement of biochemical data (catecholamine types) as well as Ki-67 indexes. Older cases without original Ki-67 data (11 MEN 2A tumors) were recut and stained with an anti-Ki-67 antibody in a clinically accredited pathology laboratory using standardized methodology. For the sporadic control group, all Ki-67 proliferation counts were retrieved from the pathology reports. The histological and biochemical results as well as clinical follow-up data for the MEN 2A cases are presented in Table 1, and examples of positive histological PASS and GAPP parameters are illustrated in Fig. 1. An Olympus BX46 microscope equipped with a ToupTek E3CMOS camera and ToupView software v. 3.7 was used for evaluation of slides and acquisition of photomicrographs. In addition, clinical follow-up of all patients was reviewed (Table 1).

**Table 1:** MEN 2A patient characteristics with PASS and GAPP scores

Clinical characteristics						Grading system for adrenal pheochromocytoma and paraganglioma (GAPP)							Pheochromocytoma of the Adrenal Gland Scaled Score (PASS)*									
Patient	Gender	Age at diagnosis	Tumor size (mm)	*RET* mutation	Follow-up time (months)*	Histological pattern	Cellularity	Comedo necrosis	Vascular or capsular invasion	Ki-67	Catecholamine type	Total GAPP	Large nests or diffuse growth	Focal or confluent necrosis	High cellularity	Cellular monotony	Tumor cell spindling	Mitotic figures	Capsular invasion	Profound nuclear pleomorphism	Hyper-chromasia	Total PASS
1A	M	24	29	c.1900T>C	17	1	1	0	0	2	0	4	2			2		2				6
1B	M	25	50	c.1900T>C	4	0	1	0	0	2	0	3					2	2				4
2A	F	41	40	c.1900T>G	+ 200	1	0	0	0	1	0	2	2							1		3
2B	F	44	30	c.1900T>G	+ 200	0	0	0	0	1	0	1										0
3A	M	41	50	c.1900T>G	+ 200	1	2	0	1	1	0	5	2		2		2		1			7
3B	M	42	40	c.1900T>G	+ 200	1	0	2	0	1	0	4	2	2						1	1	6
4	M	53	40	c.1900T>G	+ 200	1	1	0	0	1	1	4	2							1		3
5	F	45	30	c.1900T>G	+ 200	1	0	0	0	1	0	2	2									2
6	F	24	25	c.1900T>C	36	1	1	0	0	2	0	4	2		2			2				6
7	M	33	20	c.1900T>G	48	1	2	0	0	2	0	5	2		2		2				1	7
8	F	34	50	c.1900T>G	+ 200	1	0	0	0	1	0	2	2									2
9	F	45	70	c.1900T>G	96	1	0	0	0	0	0	1	2								1	3
10	F	30	30	c.1858T>C	84	0	1	0	0	1	0	2			2							2

None of the tumors showed atypical mitotic figures, periadrenal adipose tissue invasion or vascular invasion

*No patient had any radiological or biochemical evidence of disease at follow-up

### Statistical analyses

Statistical analyses were made with IBM SPSS Statistics 24 (IBM, Armonk, NY, USA). Non-normal distribution was assumed for all data, applying the Mann-Whitney *U* and Fisher’s exact tests for comparison between groups. *P* values < 0.05 were considered as statistically significant.

## Results

The median age was 41 for the MEN 2A group and 63.5 for the sporadic controls. The MEN 2A group consisted of 7 female and 6 male patients, whereas the sporadic controls included 16 female and 12 male patients. The majority of MEN 2A patients in this series have been followed for over 200 months and were all free of metastatic or recurrent disease (Table 1). No recurrences or metastases were observed in the sporadic group, and the follow-up time ranged from 1 to 82 months (40 months on average, median 38 months).

In total, six PCCs from four MEN 2A patients exhibited a PASS score ≥ 4 (ranging from 4 to 7), and these tumors were thereby classified as exhibiting “potential for aggressive behavior” (Table 1). The remaining seven tumors exhibited scores from 0 to 3. Hence, 6/13 MEN 2A-related tumors (46%) were exhibiting malignant potential by using the PASS algorithm (Fig. 1). The most prominent positive factor was the finding of large nests/diffuse growth in 10/13 PCCs (77%), followed by “high cellularity” (seen in 4/13; 31%) (Table 1). In contrast, 4/28 (14%) sporadic PCCs demonstrated PASS scores ≥ 4 (Fig. 1). The difference between the sporadic and MEN 2A groups was statistically significant using Fisher’s exact test (*P* = 0.049) as well as the Mann-Whitney *U* test (*P* = 0.002).

The GAPP scores ranged from 1 to 5, on an average of 2.39. Using the GAPP algorithm, seven out of 13 (54%) MEN 2A PCCs displayed GAPP scores ≥ 3, thereby establishing them as “moderately differentiated” with a significant risk of future relapse or metastatic disease (Table 1, Fig. 1). The remaining five MEN 2A PCCs were “well differentiated.” All PCCs with PASS scores ≥ 4 were also “moderately differentiated” according to GAPP (Table 1). Among the sporadic controls, 9/28 (32%) scored ≥ 3 whereas the majority (19/28; 68%) displayed scores below 3 and thereby classified as “well differentiated” (Fig. 1). In this group, GAPP scores ranged from 0 to 5, with an average score of 2. The difference in GAPP scores between the sporadic and MEN 2A groups was not statistically significant using Fisher’s exact test (*P* = 0.3) nor the Mann-Whitney *U* test (*P* = 0.09). Of note, 4 out of 13 (31%) MEN 2A PCCs displayed Ki-67 counts of > 3% (earning two GAPP points) as compared to 3 out of 28 (11%) in the sporadic control group (Fisher’s exact test, *P* = 0.18).

The mean tumor sizes were 39 and 44 mm for the MEN 2A (Table 1) and sporadic group respectively. No associations were seen between tumor size and the presence of necrosis, which could signify these parameters as independent. Moreover, no associations were seen between the patient’s *RET* genotype and PASS or GAPP scores (Fisher’s exact test, *P* = 0.08 and *P* = 0.13 respectively).

## Discussion

MEN 2A patients have an approximately 50% lifetime risk of developing PCC [1]. MEN 2A PCCs are generally benign, although few reports of malignant PCCs in the setting of MEN 2A exist, with an estimated prevalence of 3% of all tumors investigated [4]. The PASS and GAPP algorithms, despite limitations in terms of reproducibility and suboptimal specificity, represent the two histological systems suggested by the current WHO classification to stratify malignant potential in PCCs. The finding of elevated PASS and GAPP scores in approximately half of the MEN 2A PCCs is intriguing, not least given the long disease-free follow-up. Interestingly, all PCCs with PASS scores ≥ 4 were also “moderately differentiated” according to GAPP, suggesting that both algorithms were concordant and most likely similarly inadequate for determining true malignant potential in MEN 2A-related PCCs.

In the original PASS publication, 8 out of 50 benign (16%) and 4 out of 50 (8%) malignant PCCs were classified as “MEN or inherited forms of pheochromocytoma” [2]. Therefore, since the vast majority of cases included were sporadic PCCs, the PASS algorithm could be suboptimal for assessing malignant potential of MEN 2A-associated cases. Regarding the GAPP algorithm, no clear-cut information regarding the percentage of hereditary PCCs enlisted is available, although the authors state that 13 out of the 163 samples (8%) assessed were bilateral, suggestive of hereditary disease. Therefore, it seems safe to conclude that neither the PASS nor the GAPP algorithms are primarily constructed for PCCs arising in hereditary settings.

The two most noticeable positive PASS factors in our MEN 2A cohort were “large nests/diffuse growth” and “high cellularity.” In the original publication, a “large nest” was defined as three to four times the size of a normal “zellballen.” Thirty percent of the benign PCCs displayed either large nests or diffuse growth, as compared to 90% of the malignant PCCs, and therefore, this parameter was weighted with a score of two points [2]. In our cohort, large nests and/or diffuse growth was observed in 77% of all MEN 2A-related PCCs. Since all our cases are thought to be benign given the absence of metastases or relapses, we believe this common phenomenon might reflect the underlying biological nature of MEN 2A-related PCCs rather than echoing a malignant phenotype. Since MEN 2A PCCs harbor *RET* proto-oncogene mutations and express a specific hypoxia gene expression signature [4], this common genetic profile might in theory result in distinct histological patterns for subsets of cases. Indeed, the effect of hypoxia on tumor growth patterns has been previously demonstrated [5].

The significant association between high PASS scores and MEN 2A PCCs was further substantiated when a similar connotation was seen when applying the GAPP algorithm. Of specific interest, MEN 2A PCCs regularly displayed Ki-67 indexes above 1%, making it the most commonly fulfilled GAPP criteria in the MEN 2A group. The association between elevated proliferation counts and MEN 2A PCCs could signify that Ki-67 itself is of less value when evaluating malignant potential in this subset of PCCs. However, it should be noted that high proliferative tumors might signify an alarming factor independently of the PASS and GAPP scores. Indeed, previous studies have pinpointed the prognostic value of Ki-67 staining in PCCs [6].

As a limitation, this study does not include cases with apparent malignant phenotypes (such as distant metastases), and therefore, we know little regarding the sensitivity of both methods in our material. In particular, the PASS algorithm has been previously shown to demonstrate reduced sensitivity, specificity, and high intra-observer variation, and several adjunct markers and clinical parameters have therefore been employed to improve the diagnostic accuracy of the method [7–13]. Some authors in particular advocate the use of SDHB immunohistochemistry as an additional tool to detect cases with potential for aggressive behavior. The majority (9/13) of MEN 2A-related PCCs in our cohort have been previously screened for somatic *SDHB* mutations, and all nine cases demonstrated wild-type sequences [14]. This would suggest that *SDHB* mutations are uncommonly found among MEN 2A PCCs and could therefore support the notion that the vast majority of these lesions are indeed benign—although endowed with high PASS and GAPP scores.

Managing MEN 2A patients can partly be based on the established genotype-phenotype correlation [15]. Patients harboring a mutation known to be accountable for development of PCC must be followed up lifelong regarding PCC, with biochemical evaluation at least yearly and adrenal imaging either based on positive biochemistry, or at individualized intervals. Whether a patient operated bilaterally for PCC must be followed up is a matter of discussion; most clinicians would at least check for biochemical evidence for recurrence, as the risk of relapse/metastases is not negligible. If a positive PASS and/or GAPP algorithm should alter the follow-up could be debated, certainly, a malignant potential in PCC usually is taken into account.

Although based on few cases, our observation suggests that the PASS and GAPP algorithms will yield many apparently false-positive cases when assessing MEN 2A-related PCCs. This is something practicing endocrine pathologists should be aware of, not least given the consequences for each individual patient—including lifelong vigilance for recurrences.
